# Spotting trends in organocatalysis for the next decade

**DOI:** 10.1038/s41467-020-17600-y

**Published:** 2020-07-29

**Authors:** José M. Lassaletta

**Affiliations:** Instituto Investigaciones Químicas (CSIC-US) and Centro de Innovación en Química Avanzada (ORFEO-CINQA), C/ Américo Vespucio 49, 41092 Sevilla, Spain

**Keywords:** Organocatalysis, Reaction mechanisms, Stereochemistry, Synthetic chemistry methodology

## Abstract

After two decades of steady growing, symbiotic merger of organocatalysis with emerging electrochemical and photochemical tools are envisioned as hot topics in the coming decade. Here, these trends are discussed in parallel to the implementation of artificial intelligence-based technologies, which anticipate a paradigm shift in catalyst design.

## The beginning and evolution of organocatalysis

Some 20 years ago, shortly after the beginning of the new millennium, the term organocatalysis was forged by Prof. David W. C. MacMillan in a paper dealing with the activation of enones by secondary amines (iminium activation) in Diels Alder reactions^1^. It was defined as “the acceleration of a chemical transformation through addition of a substochiometric amount of an organic compound which does not contain a metal atom*”*. This paper and a parallel seminal publication by List et al.^2^, dealing with the use of proline as a catalyst for direct aldol condensations (enamine activation), ignited the extraordinary blossoming of the field in the following decade. Actually, this term was immediately and unreservedly embraced by scientists working in areas as diverse as H-bonding catalysis, *N*-heterocyclic carbene catalysis, oligopeptide (foldamers) catalysis, Brønsted acid catalysis, and ion-pairing catalysis, among others. As in the case of the catalytic iminium and/or enamine activation (aminocatalysis), the simultaneous flourishing of all these areas during the decade of the 2000s contributed to a perfect storm that eventually originated the phenomenal surge of organocatalysis as one of the fastest growing topics in Chemistry.

The operational simplicity of the organocatalytic reactions, often water and air tolerant, the robustness/stability of the catalysts, their non-toxic nature, their broad functional group tolerance, and the extraordinary diversity of small organic molecules available as catalysts are valuable aspects that attracted the interest of so many working groups around the world. Additionally, many organocatalysts are easily obtained from chiral natural sources (amino acids, alkaloids, etc.) and, consequently, a considerable portion of the contributions in organocatalysis focuses on enantioselective transformations. After the initial explosive expansion, the following years witnessed the consolidation of the field with the development of concepts such as bifunctional catalysis^3^, biomimetic cascade reactions^4^, the implementation of open-shell reactivity (SOMO activation)^5^, the introduction of organosuperbases for the activation of weakly acidic nucleophiles^6^, or the activation by frustrated Lewis pairs (FLPs)^7^, among others, and the discipline passed also the test as a key tool in challenging total syntheses^8^. The evolution of the field was quantified in 2008^9^ as follows: the word organocatalysis or its derivatives had appeared in ca. 600 publication titles (Web of Science) and 40,000 web pages (indexed in Google). Today, these numbers have grown exponentially up to ca. 5000 publications and 1,260,000 web pages, which reflects a steady growing and diversification of the field, far beyond its origins.

In this immense thematic scenario, it is certainly difficult to make a prospective analysis for the starting decade. Hence, the intention of this comment is to put the focus in a short number of emerging strategies. This selection, necessarily very restrictive, includes transformations that have been traditionally accomplished using transition metal catalysis and highlight synergies that will surely have a significant impact in the near future.

## Towards new activation modes

**Fig. 1:**
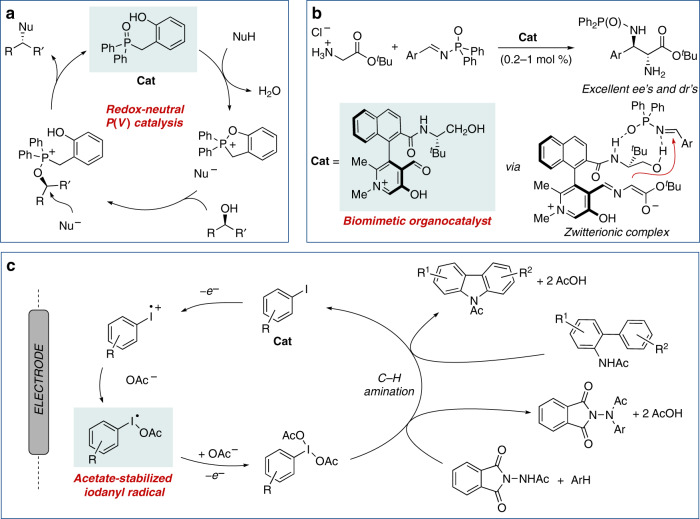
Selected innovative strategies in organocatalysis. **a** Proposed redox-neutral catalytic cycle for the Mitsunobu reaction. Nu nucleophile. **b** Biomimetic approach for Mannich reaction of free glycinates using an axially chiral pyridoxal catalyst. **c** Electroorganocatalytic C–H amination of arenes mediated by hypervalent iodine.

The high diversity of possible interactions resulting in an original organocatalytic substrate activation continuously generates new ideas and designs for specific catalytic transformations. The following couple of examples illustrate that the creativity in this field is far from fading and provides inspiring solutions for relevant chemical problems. For instance, the approach developed by Denton and co-workers^10^ for the catalytic Mitsunobu-like reaction is impressive for its simplicity. Using a specially designed dehydratable phosphine oxide organocatalyst, the long-dreamed waste-free nucleophilic substitution of secondary alcohols is cleanly performed according to the catalytic cycle shown in Fig. 1a, with H_2_O being the sole by-product of the reaction. The methodology was applied to a variety of C–O and C–N bond formation reactions in complex molecules and, using TfOH as a co-catalyst, could be also applied to ether synthesis via catalytic generation of alkyl triflates.

In a second remarkable example, Zhao’s group has recently developed an original biomimetic strategy for the challenging α-functionalization of unprotected glycinate^11^. Upon formation of enolizable imine intermediates, an N-quaternized axially chiral pyridoxal catalyst, reminiscent of the activation mechanism used by vitamin B6, enabled a highly enantio- and diastereo-selective Mannich reaction with aryl *N*-diphenylphosphinyl imines (Fig. 1b).

## Electrocatalytic transformations based on hypervalent iodine

An additional topic that has the potential to grow rapidly in the near future is the redox organocatalysis based on iodine, phosphorus or sulfur derivatives, which have the ability to mimic transition-metal-like catalysis in certain cases. Specifically, organocatalytic oxidations based on the I(I/III) redox couple have shown an extraordinary potential in organic synthesis, that has been progressively extended to include asymmetric transformations^12^. Although this is a well-established methodology, the symbiotic merger with electrochemistry offers great opportunities for further development. In a very recent study, Powers and co-workers have demonstrated that electrochemical oxidation of aryl iodides generates acetate-stabilized I(II) iodosyl intermediates, which are further oxidized to hypervalent iodine species. This process has been coupled with oxidative C–H/N–H coupling for the development of an electrocatalytic amination of arenes (Fig. 1c)^13^. Obviously, the electrocatalytic generation of I(III) species holds great potential for many other oxidation processes.

## Photo-organocatalysis

**Fig. 2:**
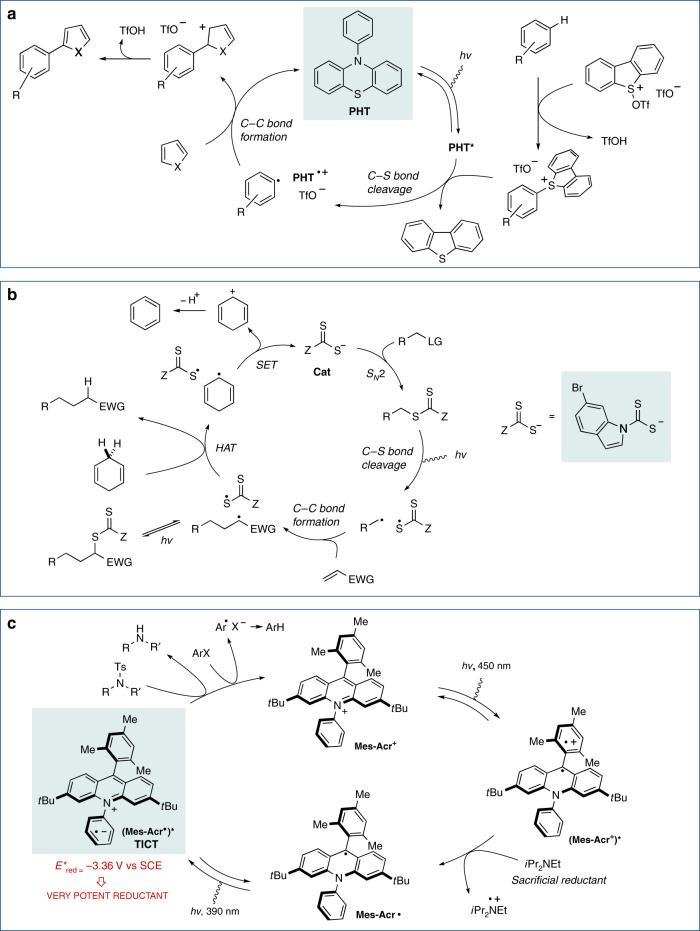
Selected photoorganocatalytic transformations. **a** Photoorganocatalytic oxidative cross-coupling of arenes and heteroarenes using an interrupted Pummerer rearrangement. **b** Photoorganocatalytic generation of free alkyl radicals: Alkylation of electrophilic alkenes. LG leaving group, SET single electron transfer, HAT hydrogen atom transfer, EWG electron-withdrawing group. **c** Photoorganoatalytic reductions mediated by an excited neutral acridine radical. TICT twisted intramolecular charge-transfer.

In 2008, a seminal publication by Nicewicz and MacMillan showed for the first time that merging photoredox catalysis with organocatalysis is a powerful tool to solve challenging chemical problems as was the direct alkylation of aldehydes^14^. After this inspirational breakthrough, the combination of photoredox activation and organocatalysis positioned itself as one of the most fruitful synergies in organic synthesis. Recently, there is an increasing interest in the development of photoactive organic compounds, aiming to complement or improve processes based on common Ir or Ru-based catalysts and develop truly metal-free processes. The following triad of beautiful examples might serve to spot the potential of this type of organocatalyst. In a first exciting and illustrative example, a genuine photoorganocatalytic approach has been used by Procter and co-workers for truly metal-free oxidative C–H/C–H cross-coupling reactions^15^, leading to elaborated (hetero)biaryl compounds from scratch (Fig. 2a). In this reaction, 10-phenylphenothiazine as the organic photoredox catalyst outperforms conventional metal-based photocatalysts, efficiently activating the intermediate aryldibenzothiophenium salts generated in an interrupted Pummerer rearrangement.

A second example taken from Melchiorre and co-workers deals with the design of an original strategy for the catalytic generation of alkyl radicals^16^. In this approach, a nucleophilic dithiocarbamate catalyst decorated with a suitable chromophore reacts in an S_N_2 fashion with electrophilic substrates to generate photoactive intermediates that are then irradiated with visible light to promote a C–S bond excision (Fig. 2b). The nucleophilic radical is then trapped by electron-poor alkenes and the catalytic cycle is closed with γ-terpinene as a cheap source of hydrogen atoms and electrons. The methodology proved to be useful in the preparation of a marketed drug, in late-stage derivatizations, and enantioselective radical catalysis.

Finally, the group of Nicewicz has recently reported on organocatalytic reductive processes based on a photoexcited neutral mesityl acridine radical (Mes-Acr^•^) featuring an extraordinary excited-state oxidation potential (−3.36 V versus a saturated calomel electrode, stronger than alkaline metals). This species, characterized as a twisted intramolecular charge-transfer (TICT) state, was generated from an acridinium catalyst after two PET events and with the help of iPrNEt_2_ as a sacrificial reducing agent (Fig. 2c). Metal-free reductive dehalogenation of aryl halides and detosylation of amines were developed as the first applications of the methodology. Remarkably, the excited mesityl acridinium salts used in this reaction is also commonly used as a potent photooxidation catalysts.

## Chemoinformatics and machine-learning techniques in organocatalysis

Finally, there is a predictable revolution that is probably going to change the field of computer-assisted catalyst design. Until now, computational theoretical techniques, from Hybrid Quantum Mechanics/Molecular Mechanics (QM/MM) approaches to unrestricted Density Functional Theory (DFT) methods, have been invaluable tools in catalysis to provide insights into the reaction mechanisms and, in limited occasions, helping to perform a fine tuning of catalysts based on stereochemical models, but failed to become a useful tool at the prediction level. Besides serendipitous findings, until now catalyst design has been driven mainly by human creativity, but in most cases the fine tuning to reach high activity/selectivity has been the result of a tedious and time-consuming trial and error practice, and in many cases a non-optimal result is achieved from the limited set of candidates considered in the screenings.

**Fig. 3:**
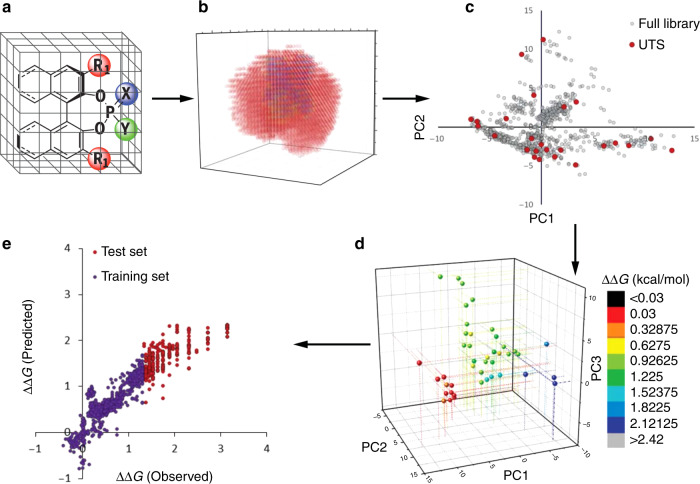
Steps for an application of chemoinformatics in organocatalyst design. **a** An in silico library of catalyst candidates is generated. **b** Relevant chemical descriptors are calculated. **c** A universal training set (UTS) is selected. **d** Experimental selectivity data are collected. **e** Machine learning predicts high-selectivity reactions using moderate-to-low-selectivity reactions. The figure content is reproduced with permission from AAAS^18^.

Today, however, artificial intelligence-based strategies, fueled by improved computing power, data availability and sophisticated machine-learning algorithms, are starting to be implemented as a useful tool in chemistry, breaking some of the previous limits in catalyst design^17^. In a very illustrative example, Denmark and co-workers have recently reported on the use of averaged steric occupancy (ASO) descriptors to accurately predict the enantioselectivities of chiral phosphoric acid catalyst in the addition of thiols to N-acylimines, according to the work-flow shown in Fig. 3 ^18^.

The contributions highlighted above are just a selection of breaking discoveries that will surely be the basis for future developments. With creativity always driving innovation forward, the synergistic combination with emerging technologies in electrochemistry, photocatalysis and chemoinformatics are envisioned as a powerful driving force for the development of organocatalysis in the coming years.
